# Modeling Reading Ability Gain in Kindergarten Children during COVID-19 School Closures

**DOI:** 10.3390/ijerph17176371

**Published:** 2020-09-01

**Authors:** Xue Bao, Hang Qu, Ruixiong Zhang, Tiffany P. Hogan

**Affiliations:** 1 School of Health and Rehabilitation Sciences, Massachusetts General Hospital, Institute of Health Professions, Boston, MA 02129, USA; 2 School of Earth and Atmospheric Sciences, Georgia Institute of Technology, Atlanta, GA 30332, USA; pku.quhang@gmail.com (H.Q.); zhangruixiong@gmail.com (R.Z.); 3 ClimaCell Inc, Boston, MA 02210, USA

**Keywords:** reading development, school closure, children, COVID-19, read to child, mitigation, book, parent–child interaction, policy making, education

## Abstract

By 15 April 2020, more than 1.5 billion students worldwide experienced school closures in an effort to slow the spread of a novel coronavirus, severe acute respiratory syndrome coronavirus 2 (SARS-CoV-2), during the worldwide coronavirus disease 2019 (COVID-19) pandemic. These interruptions in formal in-person educational experiences caused adverse consequences on school-age children’s academic outcomes. Using a pre-existing database, we calculated changes in children’s reading ability without formal education (i.e., the summer months). The resultant models predicted that the rate of reading ability gain in kindergarten children during COVID-19 school closures without formal in-person education will decrease 66% (2.46 vs. 7.17 points/100 days), compared to the business-as-usual scenario, resulting in a 31% less reading ability gain from 1 January 2020 to 1 September 2020. Additionally, the model predicted that kindergarten children who have books read to them daily would have 2.3 points less loss (42%) compared to those who do not, who are predicted to have a 5.6-point loss during the same time period. Even though reading books to children will not substitute the critical role of formal education in teaching children how to read, families, educators, and policy makers can promote this simple strategy to facilitate and maintain reading ability gain during school closures, which may be a common occurrence as nations see the public health benefits of physical distancing for the current and future pandemic outbreaks.

## 1. Introduction

To stop the spread of a novel coronavirus, severe acute respiratory syndrome coronavirus 2 (SARS-CoV-2), during the coronavirus disease 2019 (COVID-19) pandemic, by 15 April 2020, 91.3% of children enrolled in formal schooling—1.5 billion children worldwide from 192 countries—were ordered to stay home [1]. The interruptions in formal in-person educational experiences adversely impacted school-age children [2]. In the United States, more than 130,000 school closings impacted almost 57 million children [3]. All fifty states in the U.S. had closed their schools for at least 3 weeks, and 49 states had closed all public and private schools for the rest of the 2019–2020 academic year [3]. In these unprecedented times, children will be out of school half as much as they were in school this academic year (approximately 92 days out of 180 days) [4].

Many countries have implemented the school closure policy as an immediate response to the COVID-19 outbreak to reduce social contact [1]. The effectiveness of school closures on the spread of the coronavirus remains unknown [5], yet the adverse consequences of school closures on children’s growth and development started to appear soon afterward [6,7,8]. Recent studies showed that children have fewer regular eating and sleeping patterns, longer screen time, fewer physical activities, increased stress, and less social interaction during school closures, which pose risk to their physical and mental health [8]. Among all the possible risks that COVID-19 school closures pose on children’s physical and mental health, the United Nation Educational, Scientific and Cultural Organization (UNESCO) listed “interrupted learning” among the top adverse consequences of COVID-19 school closures [1]. Formal schooling—in person or remote—provides essential knowledge and skills for children’s growth and development [1]. Therefore, the interruption of formal education may cause a negative impact on children’s learning outcomes, especially for disadvantaged children with unequal access to educational resources [1].

The effect of school closure on academic achievement has been studied in the summer months. This well-documented “summer slump” shows that children lose the equivalent of approximately one month of reading abilities when they are out of school during the summer break [9]. The slump in reading achievement is reduced by attending summer schools, visiting libraries, and/or participating in reading-rich summer-based activities [9,10,11,12,13]. During the 2020 COVID-19 school closures, these activities were not available to children, so there is a strong motivation to determine options that will facilitate and maintain reading development.

In the current study, we use previous kindergarten and 1st grade reading scale scores in the U.S. to predict the reading ability gain in kindergarten children during COVID-19 school closures, using data from a large nationally representative longitudinal dataset collected by the National Center for Educational Research within the Institute of Education Sciences of the U.S. Department of Education. This dataset included children and families from diverse socioeconomic backgrounds and racial/ethnic groups. One recent study used past data to predict learning rates in reading and math during COVID-19 school closures in 3rd–7th grade children in the U.S. [14]. They found that students only gained 63%–68% of grade-specific reading skills during the 2019–2020 spring semester because of the interruption of the semester [14]. In our study, we focus on the impact of school closures on kindergarten children, because children’s formal education experience begins at kindergarten, and, as such, children learn critical reading abilities then, such as phonological awareness, language structures, word decoding, and comprehension, which set the foundation for the future acquisition of reading abilities [15,16].

Furthermore, because of school closure and stay-at-home policies, children are spending more time at home with their parents than they normally have [8]. While staying at home may offer increased time to strengthen family bonds, parents are also facing more challenges parenting full-time [8]. For example, they are not only ensuring their children’s health and safety but also monitoring their physical and emotional development and facilitating learning at home [17]. Of note, the previous study on the loss of academic achievement during COVID-19 school closures did not provide evidence in the effectiveness of alternatives to in-person education, such as home schooling and remote learning or maintenance strategies such as book reading [14].

Previous studies showed that children from different socioeconomic status have different reading achievement during summer breaks [9,10]. Children from high socioeconomic backgrounds gained more reading abilities during summertime than children from low socioeconomic backgrounds. In this study, we are interested in whether these differences persist in COVID-19 school closures; therefore, we modeled the rates of reading ability gain in kindergarten children in families with different socioeconomic backgrounds. Our results may inform policy makers, educators, and families as they make decisions about the maintenance of children’s reading abilities during school closures, a likely regular occurrence for formal education until the COVID-19 pandemic comes to an end.

## 2. Materials and Methods

### 2.1. Data and Sampling

The data used to predict the reading ability gain in kindergarten children during COVID-19 school closures in this study were drawn from the Early Childhood Longitudinal Study, Kindergarten Class of 2010–2011 (ECLS-K:2011), which was a longitudinal study of 18,170 children who started kindergarten in the 2010–2011 school year from both private and public schools in the U.S. The same children were followed from kindergarten to fifth grade (2015–2016 school year). Data from multiple sources (e.g., child, family, school, and community) were collected through direct assessments, interviews, and questionnaires. Multistage sampling design was used to determine a nationally representative sample [18,19]. The full description of the dataset can be found in ECLS-K: 2011, Kindergarten Psychometric Report and 1st grade Psychometric Report [18,19].

In this study, we included all children (*n* = 3657) with complete information on parents’ responses on book reading frequencies and reading scale scores taken during kindergarten fall, kindergarten spring, 1st grade fall, and 1st grade spring (Table 1). In all, 52% of the sample were males and 48% of the sample were female [20]. Moreover, 85.1% of the sample started kindergarten at the age of five, which is the typical entering age for kindergarten in the U.S. [20]. Furthermore, 6.7% of the sample started kindergarten before the age of five, and 6.8% of the sample started kindergarten after the age of six. Demographic information was collected in the kindergarten fall semester parent questionnaire. Parents’ race/ethnicity and highest education level are described in Table 1. The questionnaire did not specify parent 1 and 2 as mother or father of the children. In the current sample, for parent 1, 43.3% were White; 7.9% were African American; 30% were Hispanic; 7.7% were Asian and Pacific Islanders; and 2.8% did not specify their race or ethnicity group. On the other hand, for parent 2, 35.7% were White; 3.9% were African American; 23.2% were Hispanic; 6.1% were Asian and Pacific Islanders; and 1.9% did not specify their race or ethnicity group. Overall, 9.7% of the parents had less than a high school diploma; 23% had high school diploma or equivalent; 21.6% had some college education but did not receive a degree; 21% had bachelor’s degree; and 17% had graduate degree. Data on home reading frequency were collected through the 1st grade fall semester parent questionnaire.

### 2.2. Reading Ability Outcome Measures

The ECLS-K:2011 used a screening test and two-stage assessment to measure reading and language abilities [18,19]. The purpose of the two-stage design was to optimize measuring accuracy and administration time. The reading assessments were based on the National Assessment of Education Progress (NAEP) Reading Framework. Each child completed a thirty-minute computer-assisted language screening and reading assessment. The language screener included two tasks from the Preschool Language Assessment Scale task (*pre*LAS 2003) that measured receptive and expressive language proficiency. Children who passed the language screening or who spoke English as their home language proceeded to the reading assessment. The reading assessment tested children’s ability in basic reading skills (e.g., phonological awareness, familiarity with print, letter/word recognition, and sight words), expressive and receptive vocabulary, and reading comprehension (e.g., recalling, interpretation, and evaluation). Language and reading assessments used in the ECLS-K:2011 besides *pre*LAS were Peabody Picture Vocabulary Test—3rd edition (PPVT-III), Test of Early Reading Ability—3rd edition (TERA-3), and Test of Preschool Early Literacy (TOPEL) [18,19].

The ECLS-K:2011 used the item analyses to calculate composite scores to quantify reading abilities, so that the scores were comparable between children [18,19]. Because this process comprised a battery of assessments of word recognition and language comprehension, we refer to the composite as “reading ability”.

### 2.3. Data Analysis

#### 2.3.1. Modeling Rates of Reading Ability Gain

We used the multivariate linear regression model with the ordinary least square method [21] to predict the rates of reading ability gain using the reading scale score from the four assessments taken during kindergarten and 1st grade (Section 2.1.). In the regression model, we used reading scale scores as the predictors and the outcome variables were the rates of reading abilities gain. Following the same assumption in previous studies predicting summer reading ability, we assumed a linear relationship between reading ability gain and time [9,10]. We used the “fitlm” function in MATLAB R2017b (The MathWorks. Inc, Natick, MA, USA) to conduct the analysis.

Here, we used y to denote the individual reading scale score (four for each participant in our sample) and *x*_1_, *x*_2_, *x*_3_ to denote the corresponding number of days in the kindergarten, summer vacation, and 1st grade, respectively. Then the linear model could be interpreted as follows:

(1) $$y_{i}=\beta_{0}+\beta_{1}x_{1i}+\beta_{2}x_{2i}+\beta_{3}x_{3i}+\varepsilon_{i}$$

where *i* represented the count of the reading scale score, *β*_0_ was the reference level of reading activity score at the first assessment (i.e., intercept of the linear model), *β*_1_ was the rate of reading ability gain in kindergarten after the first assessment, β_2_ was the rate of reading ability gain during the summer vacation between kindergarten and 1st grade, *β*_3_ was the rate of reading ability gain during the 1st grade, *ε*_*i*_ was a random error. We further denoted $X=\left[1,x_{1},x_{2},x_{3}\right]$ and $\beta ={\left[\beta_{0},\beta_{1},\beta_{2},\beta_{3}\right]}^{\prime }$, then the linear model could be interpreted as

(2)  y=Xβ+ε

**Figure 1:**
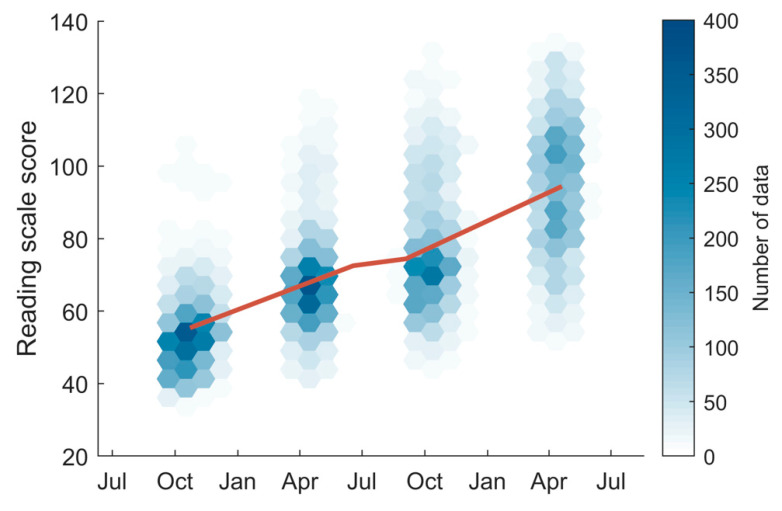
The number densities of reading scale scores are shown as the colored hexagons. The red line represents the fitted reading scale score gain using all available data.

The corresponding ordinary least square estimation of the rates of reading ability gain and corresponding covariance matrices were

(3) $$\hat{\beta }={\left(X^{\prime }X\right)}^{-1}X^{\prime }y$$

(4) $$Var\left(\hat{\beta }\right)={\left(X^{\prime }X\right)}^{-1}{\hat{\sigma }}^{2}$$

where ${\hat{\sigma }}^{2}$ were the mean of square of error $\frac{1}{m-1}{\left\|y-X\hat{\beta }\right\|}^{2}$, and m was the total number of assessment scores. The distribution of the reading scale scores in the four assessments and the fitting results are shown in Figure 1.

#### 2.3.2. Differences between Rates of Reading Ability Gain

The differences between the rates of reading ability gain during summer and kindergarten $\left(\beta_{2}-\beta_{1}\right)$ and the differences between the rates of reading ability gain during summer and 1st grade $\left(\beta_{2}-\beta_{3}\right)$. were then calculated using the corresponding vectors $a_{1}=\left[0,-1,1,0\right]$ and $a_{2}=\left[0,0,1,-1\right]$ such that $a_{1}\beta =\beta_{2}-\beta_{1}$ and $a_{2}\beta =\beta_{2}-\beta_{3}$. Let $z_{1}=a_{1}\beta$, and $z_{2}=a_{2}\beta$, we then have

(5) $${\hat{z}}_{1}=a_{1}\hat{\beta }$$

(6) $$Var\left({\hat{z}}_{1}\right)=a_{1}Var\left(\hat{\beta }\right)a_{1}^{\prime }$$

(7) $${\hat{z}}_{2}=a_{2}\hat{\beta }$$

(8) $$Var\left({\hat{z}}_{2}\right)=a_{2}Var\left(\hat{\beta }\right)a_{2}^{\prime }$$

Furthermore, let $t_{1}=\frac{{\hat{z}}_{1}}{\sqrt{Var\left({\hat{z}}_{1}\right)}}$ and $t_{2}=\frac{{\hat{z}}_{2}}{\sqrt{Var\left({\hat{z}}_{2}\right)}}$, *t*_1_ and *t*_2_ should both follow a t-distribution with degrees of freedom of *n*−4 and we can then derive the *p*-value from the t-distribution.

#### 2.3.3. Predicting Rates of Reading Ability Gain from Different Reading Frequencies

To predict the effects on the rates of reading ability gain from different home reading frequencies, we partitioned the data by five factor categories namely “never read to child”, “once or twice a week”, “three to six times a week”, “not every day”, and “every day”, and calculated the rates of reading ability gain in each partition of the data, respectively, as follows:

(9) $$y_{ij}=\beta_{0j}+\beta_{1j}x_{1ij}+\beta_{2j}x_{2ij}+\beta_{3j}x_{3ij}+\varepsilon_{ij},\;\;j=0,1,2,\ldots$$

(10) $$y_{j}=X_{j}\beta_{j}+\varepsilon_{j},\;j=0,1,2,\ldots$$

where j represented each one of the five factor categories.

## 3. Results

### 3.1. Modeling the Rate of Reading Ability Gain during COVID-19 School Closures

We used the Early Childhood Longitudinal Study, Kindergarten Class of 2010–11 (ECLS-K:2011) data [18,19] to model the effect of school closures on the rates of reading ability gain of kindergarten children. Using a multivariate linear regression model (described in Section 2.3.1.), we predicted the rate of reading ability gain, measured by reading scale scores during kindergarten spring semester as $\beta_{1}=7.17\pm 0.18$ points per 100 days across the nation (Table 2). During the summer vacation, children received no formal education from schools and the rate of reading ability gain decreased to $\beta_{2}=2.46\pm 0.51$ points per 100 days, significantly slower than the rate during kindergarten and 1st grade (*p* < 0.001, Section 2.3.2). Assuming that the rate of reading ability gain of kindergarten children during COVID-19 school closures is the same as that during the summer vacation between kindergarten and 1st grade (i.e., *β*_2_), the predicted rate of reading ability gain would decrease from 7.17 by 66% to 2.46 points per 100 days during COVID-19 school closures.

**Figure 2:**
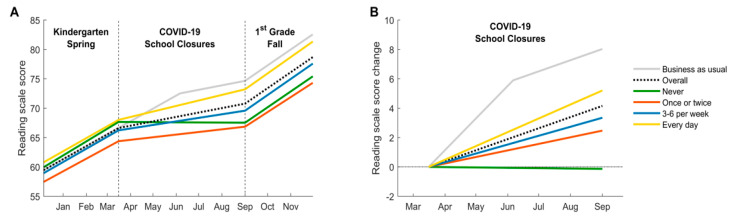
The predicted reading scale scores before, during, and after COVID-19 related school closures. The gray line in Panel A shows the anticipated reading scale score in the business-as-usual semesters with formal education. The dotted, green, orange, blue, and yellow lines represent the anticipated reading scale score (Panel **A**) and the score change (Panel **B**) with COVID-19 school closures of all children in the sample, children whose parents never read to them or read once or twice a week, three to six times a week, and every day, respectively. Panel A shows the predicted reading scale score changes over time during 2020 kindergarten spring semester, COVID-19 school closures, and 2020 1st grade fall semester. Panel B shows the predicted change of reading scale score since 16 March 2020 during school closures of children whose parents read to them at different frequencies.

Without COVID-19 school closures, the anticipated averaged reading ability in kindergarten children would improve by 13.8 points from 1 January to 1 September 2020 (the approximate start of 1st grade fall semester). To control the spread of COVID-19, most of the states in the U.S. have enforced school closures starting the week of March 16th. Assuming that kindergarten children did not receive effective alternatives of formal education since then, the anticipated averaged reading ability gain for these kindergarten children (dotted line in Figure 2A) would change to 9.5 points from 1 January 2020 to 1 September 2020, 31% less than that without COVID-19 school closures (grey line in Figure 2A).

### 3.2. Anticipated Effect of Reading Books to Children Every Day on Reading Ability Gain

Many activities proven to support and maintain reading ability gain during school closures—attending summer schools, visiting libraries, and doing summer reading activities—were not available during the COVID-19 closures [9,10,11,13]. Therefore, we investigated the effect of reading books to children at home on reading ability gain, an activity that can be accomplished during COVID-19 closures. The ECLS-K:2011 dataset included parents’ estimates of how frequently they read to their child during the summer months. They were given four options in the questionnaire: not at all, once or twice a week, three to six times a week, and every day. Results from the linear regression model (Section 2.3.3) predicted that the more frequently children were read to, the more reading abilities they gained during summertime school closures (Table 2 and Figure 2). Children whose parents read to them every day had the highest anticipated rate of reading ability gain of $\beta_{2}=3.08\pm 0.78$ points per 100 days, 79% higher than the children whose parents could not do so ($\beta_{2}=1.72\pm 0.67$ points per 100 days) (Table 2 and Figure 2B). Children whose parents never read to them had the lowest anticipated rate of reading ability gain ($\beta_{2}=-0.08\pm 4.19$ points per 100 days, note that the large variability was due to its small sample size).

Based on these results, the model predicted that children whose parents read to them every day will gain 10.6 points from 1 January to 1 September 2020 with COVID-19 school closures. In comparison, children whose parents cannot read to them every day and those whose parents never read to them will gain 8.3 and 5.2 points during the same time period, respectively (Figure 2). Compared to the business-as-usual scenario without COVID-19 school closures, children whose parents read to them every day would gain 3.2 points less while those whose parents cannot do so would gain 5.5 points less. This suggests that reading books to kindergarten children every day may mitigate about 42% (or 2.3 points) of the potential loss in reading ability gain.

### 3.3. Modeling Rate of Reading Ability Gain on Kindergarten Children from Families in Different Socioeconomic Backgrounds

To evaluate the effect of COVID-19 school closures on kindergarten children from families in different socioeconomic backgrounds, we further divided our sample into four subgroups based on their family income and parents’ highest education level (i.e., low family income + low education level, low family income + high education level, high family income + low education level, and high family income + high education level). The cutoff we used for family income and parents’ highest education level were above 185% federal poverty level and at least one parent with a bachelor’s degree, respectively. Using the same method described in Section 2.3.3, we predicted that children with low family income and low parents’ education level would have the highest rate of reading ability gain ($\beta_{2}=3.32\pm 0.73$ points per 100 days) during COVID-19 school closures, whereas children from high income and high education families would have the lowest rate of reading ability gain ($\beta_{2}=3.32\pm 0.73$ points per 100 days) (Table 3). Directly comparing these rates of reading ability gain may be subject to uncertainties, because of the large difference in the baseline scores (*β*_0_). Children from high income and high education families had a baseline score that was 10.6 points higher than what children from low income and low education families had. These predictions suggested that the large gap between the reading abilities of these two subgroups with different socioeconomic backgrounds might be filled after roughly 530 days of school closures without formal education. The anticipated rates of reading ability gain in children from the rest of the four groups (low family income + high education level and high family income + low education level) stayed between the other groups (i.e., high family income + high parent education level and low family income + low parent education level), namely $\beta_{2}=3.16\pm 2.35$ and $\beta_{2}=2.97\pm 1.00$ points per 100 days, respectively (Table 3). However, the relatively small sample size in these two groups caused large variability in the estimates.

## 4. Discussion

We used data from a large nationally representative longitudinal study to model reading gains during COVID-19 school closures. The model predicted that the rate of reading ability gain in kindergarten children will decrease 66% during COVID-19 school closures compared to what they would normally have in the business-as-usual scenario. Our results are consistent with previous studies on summer slump [9]. Cooper et al. revealed that, at best, students showed no loss during summer, but at worst, they lost one-month of grade level equivalent reading abilities [9].

Two recent studies predicted the impact of COVID-19 school closures on academic outcomes in various subject, grades, and countries from different perspectives using data collected before COVID-19 school closures [14,22]. Kuhfeld et al. showed that 3rd–7th grade students will lose approximately 35% of reading gains compared to what they would have gained in a typical school year [14]. They suggested that the loss in reading ability is unbalanced among students, with the top students losing less [14]. Azevedo et al. predicted academic outcome from a financial perspective in different school closure scenarios (i.e., schools closed for 3, 5, and 7 months with various levels of effectiveness of the mitigation strategies) [22]. They concluded that when schools are closed for 5 months, primary and secondary school students in the current cohort will lose on average ~$900 yearly income individually, which equals to a present value of $10 trillion worldwide [22]. They suggested that governments should take actions to build and implement safe and effective post-COVID education plans to ensure the continuity of the formal education in schools or at home through robust remote learning [22].

Past studies have shown that the reduced rate of reading ability gain over the summer can be mitigated by educational efforts such as intensive summer instructions and frequent library visits [9,10,11,12,13]. However, because of the COVID-19 closures, many options of formal education are not available. On the other hand, reading to children daily is a low-tech strategy to prevent some of the adverse consequences of COVID-19 school closures while strengthening family bonds. Kindergarten children, who have limited ability to read independently, depend on adults to provide access to books through read-alouds. These read-alouds provide “book-language”, which is higher in sentence complexity and school vocabulary than conversations and oral storytelling [23,24]. Reading aloud also provides an opportunity to practice word reading and interaction with text around letters and sounds, even if reading the same book multiple times [25]. In this study, our model showed that reading to young children every day helped compensate some of the anticipated reading loss during COVID-19 school closures. Our recommendation of reading to children is supported by previous studies, which showed that book reading can mitigate summer slump [12,25]. Of note, children need to master the ability to read words and the language comprehension skills to understand those words [26]. Reading to kindergarten-age children will likely have a stronger effect on a child’s language comprehension compared to early word reading, which requires comprehensive and explicit formal instruction (either in person or online) by a competent educator, especially for children who struggle to attain early reading skills [27]. Reading books to children at home is not a long-term solution for teaching reading to kindergarten children; it cannot substitute the critical role of formal education—in person or remote [27]. However, during school closures, our model predicted that reading daily to a child may mitigate some anticipated decreases in reading abilities.

The interruption of formal education has caused unprecedented pressure and frustration in families, especially those already dealing with economic hardships [28,29]. Past studies show that parents’ ability to read to their children is impeded by limited access to books and to one-on-one reading time, especially in low-income families [30,31]. Because of these barriers, we call for policy improvements and community support to ensure children have access to books and a supportive adult, not only a parent, to read to them during COVID-19 school closures. For example, studies showed that sending as few as four to five books home can improve children’s reading abilities, because every additional book in a home library may help children gain more reading opportunities in the future [30,31] Another study revealed that 15–20 min of reading time every day or at least several times a week promoted a culture of reading at home and benefited children’s growth in reading ability [32]. Some examples of local, state, and federal government include (1) providing virtual or remote library access to families during COVID-19 shutdowns, especially in low-income neighborhoods, (2) promoting book giveaway programs, (3) raising public awareness about the negative impact of the interruption of formal education on children’s reading abilities, and (4) encouraging parents to use limited time with children to read books to them [30,33]. To alleviate some parenting pressure, many local school districts, companies, and organizations are providing free audio books and remote story reading to children [34,35,36]. Some activities, such as offline audio storybooks and cellphone-based programs are especially built for children without internet and computer access [37].

There are two limitations worth noting in the current study. First, previous studies have shown that the interruption of formal education has an unequal effect on children with or without disadvantages (e.g., poverty and disability) [22,38,39]. In this study, we calculated the rates of reading ability gain in different socioeconomic background subgroups. Our results showed that children from high socioeconomic background families had a slower rate of reading ability gain compared to children from low socioeconomic background families, which contradicted previous studies [9,10]. The large difference between the intercepts of these two groups might cause large uncertainties in our results and the subsequent discrepancies compared to previous studies. Therefore, to provide a big picture and unbiased predictions of the impact of school closures, our main analysis included kindergarten children from all socioeconomic backgrounds in the sample. Future research is needed to examine the degree of impact of COVID-19 school closures on children living in all types of socioeconomic backgrounds.

Second, since the start of school closures, parents and educators are actively seeking the best way to continue formal education [40]. In all, 83% parents in the U.S. reported that their children received remote learning programs from their schools during COVID-19 closures [41]. It is widely believed that remote learning is the most effective alternative to traditional in-person schooling [42]. However, we excluded the effect of remote learning in predicting the rate of reading ability gain during COVID-19 school closures. The main reason we excluded this factor was that the dataset we used for our main analysis did not have information regarding remote learning, and we did not have enough quantitative evidence to examine the impact of remote learning in reading ability gain in kindergarten children. Because of the exclusion of the impact of remote learning, the actual reading ability gain during COVID-19 school closures may be different from our predictions. The effectiveness of remote learning on children’s reading abilities during COVID-19 school closures remains unknown until we measure children’s reading ability before, during, and after the COVID-19 school closures. We call for research studies that investigate the rate of reading ability gain during COVID-19 school closures, especially in comparing reading abilities in children who receive different formats and intensity of remote learning.

Of note, parents and teachers reported that students’ engagement in remote learning was relatively low, especially in young learners [28]; in fact, only 60% of students participated or engaged in remote learning regularly in the U.S. [28]. In addition, the design and delivery of effective or partially effective remote learning largely depended on factors such as education quality, teachers’ experiences, government/policy support, community support, access to internet, computer, television, radio, social media, and electricity [42]. According to the United Nations Children’s Fund (UNICEF), more than half of the children worldwide do not have access to these recourses [42]. Even with sufficient resources, some school districts still chose to end their school year early due to the difficulties they faced in providing remote education and the low participation rate [29]. Many other studies have reported on the pros and cons of remote learning [40,43,44]. Future research is needed to quantify the effectiveness of other alternatives such as online distant learning provided by schools, homeschool, hybrid courses, informal online learning programs, tutoring, paid private education, etc.

With an anticipated “second-wave” of COVID-19 cases, many states in the U.S. have slowed down or paused their reopening plans [45]. Our study predicted that kindergarten children would generally have 31% loss of reading ability gain from January 1st to September 1st, 2020, assuming schools will reopen in the fall semester. However, if schools remain closed or reopen partially, based on our current predictions, the adverse effect of school closures will be more pronounced. Formal schooling through active online learning will likely yield the greatest maintenance of reading abilities but until schools have robust, evidence-based systems for delivering high-quality online learning, reading to children may be an effective low-tech strategy that is predicted to help maintain kindergarten children’s reading ability and promote a love of books and social–emotional connections between parents and children.

## 5. Conclusions

In an effort to slow the spread of COVID-19, 192 countries ordered school closures, which impacted more than 1.5 billion students worldwide [1]. In our study, we used longitudinal data to quantify reading ability gains during COVID-19 school closures. We predicted that kindergarten children will gain 66% slower in their reading ability during COVID-19 school closures than they would have without school closures. We anticipated that the average gain of reading score in kindergarten children during 1 January 2020 to 1 September 2020 will decrease from 13.8 to 9.5 by 31% for lack of formal education. Our analysis showed that reading books to children every day can mitigate 42% of the predicted loss. Reading books to children is commonly used as a facilitating strategy of reading ability development [22]. We recommended this low-tech yet effective strategy to alleviate some of the adverse consequences of school closures while promoting a love of books and social-emotional connections between parents and children. Our results may inform policy makers, educators, and parents as they make decisions about how best to support reading abilities during school closures, a likely staple of formal education until the COVID-19 pandemic comes to an end.

## Figures and Tables

**Table 1:** Demographic information of the current sample.

Demographic Category	*n* in Current Sample	Percentage (%)
Gender		
Male	1901	52.0
Female	1756	48.0
Age entering kindergarten (in years)		
≤5	245	6.7
5–6	3112	85.1
>6	249	6.8
Parent race and ethnicity		
Parent 1		
White	1585	43.3
African American	288	7.9
Hispanic	1096	30.0
Asian and Pacific Islanders	281	7.7
Others	102	2.8
Parent 2		
White	1305	35.7
African American	144	3.9
Hispanic	847	23.2
Asian and Pacific Islanders	223	6.1
Others	70	1.9
Parents’ highest education		
Less than a high school diploma	355	9.7
High school diploma or equivalent	840	23.0
Some college, no degree	790	21.6
Bachelor’s degree	767	21.0
Graduate degree	622	17.0
Total	3657	100

**Table 2:** Results of the multivariate linear regression model that predicted the rates of reading ability gain during kindergarten, summer/COVID-19 school closure, and 1st grade.

Book Reading Frequency	*n* Count	*β*_0_ Intercept	*β*_1_ Rate during Kindergarten (Score Per 100 Days)	*β*_2_ Rate during Summer (Score Per 100 Days)	*β*_3_ Rate during 1st Grade (Score Per 100 Days)
Never	86	55.5 ± 1.9	7.71 ± 1.40	−0.08 ± 4.19 *	8.72 ± 1.38
1–2 times/week	752	53.5 ± 0.5	6.92 ± 0.38	1.46 ± 1.09 *	8.25 ± 0.37
3–6 times/week	1230	54.8 ± 0.4	7.28 ± 0.30	1.98 ± 0.86 *	8.85 ± 0.29
Not every day	2068	54.3 ± 0.3	7.17 ± 0.23	1.72 ± 0.67 *	8.62 ± 0.23
Every day	1589	56.7 ± 0.4	7.22 ± 0.28	3.08 ± 0.78 *	8.98 ± 0.27
All	3657	55.4 ± 0.2	7.17 ± 0.18	2.46 ± 0.51 *	8.75 ± 0.18

* The predicted differences of the rates of reading ability gain between kindergarten and summer and between summer and 1st grade are significant with *p* < 0.001 (Section 2.3.2.).

**Table 3:** Predicted rates of reading ability gain during kindergarten, summer (COVID-19 school closure), and 1st grade categorized by family socioeconomic background.

Family Socioeconomic Background	*n* Count	*β_0_*Intercept	*β_1_*Rate during Kindergarten (Score Per 100 Days)	*β*_2_ Rate during Summer (Score Per 100 Days)	*β_3_*Rate during 1st Grade (Score Per 100 Days)
Low income + low education	1307	50.8 ± 0.3	6.41 ± 0.25	3.32 ± 0.73*	7.99 ± 0.25
Low income + high education	167	55.3 ± 1.1	7.38 ± 0.81	3.16 ± 2.35	8.55 ± 0.81
High income + low education	678	54.8 ± 0.5	7.03 ± 0.35	2.97 ± 1.00*	9.20 ± 0.35
High income + high education	1222	61.4 ± 0.4	7.87 ± 0.32	1.31 ± 0.92*	9.84 ± 0.31

* The differences of the rates of reading ability gain between kindergarten and summer and between summer and 1st grade are significant with *p* < 0.001 (Section 2.3.2.).
